# Tumor Lysis Syndrome Induced by Hormonal Therapy in Metastatic Prostate Cancer: A Case Report

**DOI:** 10.7759/cureus.98643

**Published:** 2025-12-07

**Authors:** Marta Machado, Margarida Mourato, Catarina Negrão, Carolina Rodrigues, João Tiago Serra

**Affiliations:** 1 Department II of Internal Medicine, Hospital Professor Doutor Fernando Fonseca, Amadora, PRT; 2 Department III of Internal Medicine, Hospital Professor Doutor Fernando Fonseca, Amadora, PRT

**Keywords:** degarelix, gonadotropin-releasing hormone antagonist, hormonal therapy, prostate cancer, tumor lysis syndrome

## Abstract

Tumor lysis syndrome (TLS) is a life-threatening oncological emergency resulting from massive cellular destruction. Although most frequently associated with chemotherapy in hematologic malignancies, TLS is uncommon in solid tumors due to their typically slower proliferation rates and treatment response.

We report a case of a 67-year-old male with a history of untreated prostate cancer diagnosed two years earlier, who presented with abdominal pain, anorexia, and weight loss. Physical examination revealed painful hepatomegaly. Laboratory tests showed a cholestatic pattern, elevated lactate dehydrogenase, and elevated prostate-specific antigen (PSA) levels. Computed tomography (CT) revealed findings suggestive of pulmonary, hepatic, and adrenal metastases, multiple mediastinal and abdominal lymphadenopathies, and osteolytic bone lesions.

Despite the relatively modest increase in PSA levels compared to the extensive disease burden, repeat prostate biopsy confirmed a high-grade prostate adenocarcinoma (Gleason score 9).

Hormone therapy with degarelix was initiated. On the third day after treatment initiation, the patient developed both laboratory and clinical features consistent with TLS, including hyperuricemia, hyperphosphatemia, hyperkalemia, and acute kidney injury (Acute Kidney Injury Network (AKIN) stage III). A mediastinal lymph node biopsy obtained via endobronchial ultrasound was subsequently performed but was inconclusive.

TLS management was initiated, which included aggressive intravenous hydration, hypouricemic therapy with rasburicase, and correction of electrolyte disturbances. No additional disease-directed therapy, namely, renal replacement therapy, was pursued, given the rapid clinical deterioration, extensive metastatic disease, and absence of feasible oncologic treatment options, with an overall poor prognosis. The patient ultimately died 17 days after the onset of TLS.

The literature consistently shows that TLS occurring after initiation of hormonal therapy, including gonadotropin-releasing hormone-axis interventions, is associated with extremely poor outcomes. In all reported treatment-related TLS cases in prostate cancer, mortality was universal despite aggressive supportive management.

This case illustrates a rare instance of TLS occurring after initiation of gonadotropin-releasing hormone antagonist therapy in metastatic prostate cancer, highlighting the need for careful risk assessment, close biochemical monitoring, and prompt recognition and management of TLS, especially in patients with high tumor burden.

## Introduction

Tumor lysis syndrome (TLS) is an oncologic emergency resulting from massive tumor cell destruction and subsequent release of potassium, phosphate, and nucleic acids, leading to hyperkalemia, hyperphosphatemia, and hypocalcemia. These electrolyte disturbances can cause acute kidney injury, cardiac arrhythmias, and neurologic complications, including seizures [1,2].

According to the Cairo-Bishop criteria, TLS is defined by the presence of at least two laboratory criteria (hyperuricemia, hyperphosphatemia, hyperkalemia, or hypocalcemia) occurring within three days before or seven days after the initiation of oncologic therapy, together with at least one clinical manifestation (acute kidney injury, cardiac arrhythmia, or seizure) [3].

The rate of malignant cell lysis increases significantly after the initiation of antitumor therapy, accounting for the predominance of therapy-related TLS. In rapidly proliferating tumors, particularly hematologic malignancies, TLS may also occur spontaneously.

TLS is uncommon in solid tumors, with approximately 130 cases reported to date. Specifically in prostate cancer, only 10 cases have been documented - three spontaneous and seven therapy-related [4-6]. All occurred in the context of metastatic disease, and only one was associated with hormonal therapy [7].

Prostate cancer may present biological features that increase susceptibility to TLS, including high tumor burden and rapid tumor dedifferentiation. Gonadotropin-releasing hormone (GnRH) antagonists produce an abrupt suppression of androgen signaling, which can trigger sudden and extensive tumor cell apoptosis. This rapid cellular breakdown can overwhelm metabolic pathways and precipitate TLS in patients with advanced disease.

We present a rare case of TLS secondary to treatment with a GnRH antagonist in a patient with advanced metastatic prostate adenocarcinoma. This case underscores the importance of careful risk stratification before initiating hormonal therapy, as well as close clinical vigilance and biochemical monitoring in patients with a high disease burden.

This case was previously presented as a poster at the European Congress of Internal Medicine, Florence, Italy, in March 2025.

## Case presentation

A 67-year-old male smoker, with a medical history of prostate adenocarcinoma (Gleason score 7) diagnosed two years earlier in another hospital, who declined further medical follow-up, and therefore was under no active oncologic therapy, presented to the emergency department with abdominal pain, anorexia, and weight loss. He denied fever, respiratory, urinary, or gastrointestinal symptoms.

On admission, physical examination revealed abdominal distension with painful hepatomegaly. Laboratory tests showed mild anemia (hemoglobin (Hb) = 12.5 g/dL), elevated cholestatic liver pattern without hyperbilirubinemia (alkaline phosphatase (ALP) = 435 U/L, gamma-glutamyl transferase (GGT) = 462 U/L, total bilirubin = 1.02 mg/dL), hyperuricemia (8.2 mg/dL), and markedly elevated lactate dehydrogenase (LDH = 3308 U/L) and erythrocyte sedimentation rate (ESR = 103 mm/h). The total prostate-specific antigen (PSA) level was also elevated (74 ng/mL). Renal function, coagulation profile, and other serum electrolytes were within the normal range upon admission. Blood cultures and HIV serology were also negative.

**Figure 1:**
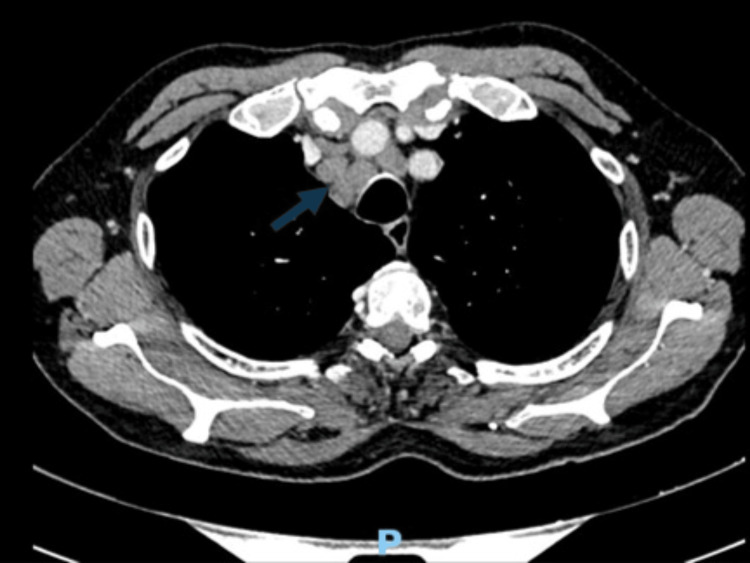
Thoracic CT showing multiple mediastinal lymphadenopathies, including a right paratracheal node measuring approximately 20 mm.

**Figure 2:**
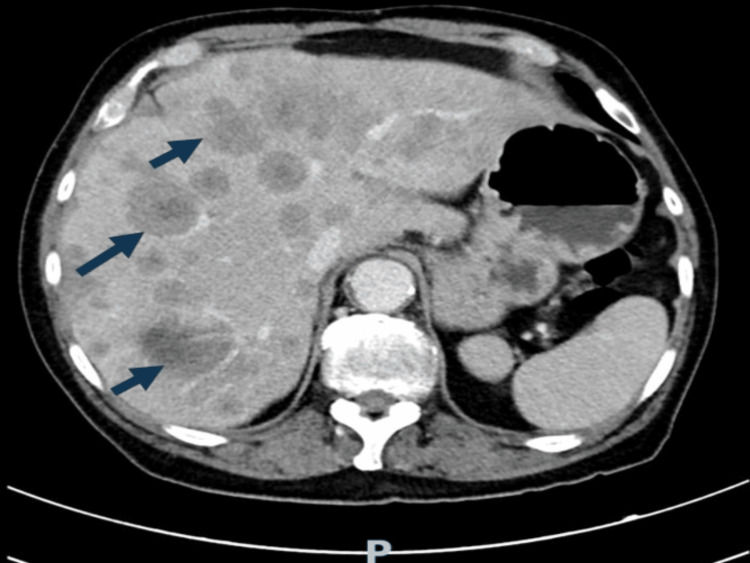
Abdominal CT scan revealing an enlarged liver with multiple heterogeneous hypodense lesions consistent with extensive hepatic metastasis.

Contrast-enhanced computed tomography (CT) of the chest, abdomen, and pelvis revealed findings suggestive of pulmonary, hepatic, and adrenal metastases, along with multiple supraclavicular, mediastinal, hilar, periaortic, and retroperitoneal lymphadenopathies. A solid pelvic mass measuring 3.5 × 3.0 cm, suggestive of metastatic implantation, was also identified, as well as osteolytic bone lesions. These findings are illustrated in Figures 1, 2.

The patient was admitted for investigation of a primary neoplasm with pulmonary, hepatic, and adrenal metastases. The most likely diagnosis considered was stage IV prostate carcinoma, despite the relatively modest increase in PSA levels compared to the extensive disease burden. During hospitalization, a prostate biopsy was performed and confirmed adenocarcinoma with a Gleason score of 9 (4+5). Mediastinal lymph node sampling was performed via endobronchial ultrasound-guided transbronchial needle aspiration (EBUS-TBNA) to characterize the primary neoplasm, targeting stations 11L, 2R, and 4R with two passes at each site. Although liver biopsy was considered, EBUS-TBNA was chosen because it allowed more immediate tissue acquisition. A PET/CT scan had also been requested, but it was not performed before the patient’s clinical deterioration. Histopathological examination of the mediastinal lymph node biopsy revealed carcinoma of indeterminate origin, with immunohistochemistry negative for PSA, racemase, thyroid transcription factor-1 (TTF-1), and melan-A. Immunophenotyping of mediastinal lymph nodes and peripheral blood was also negative. Despite the inconclusive immunohistochemical profile, dedifferentiated metastatic prostate carcinoma was considered the most likely diagnosis based on clinical and radiologic correlation.

Hormonal therapy with degarelix was subsequently initiated (240 mg subcutaneously) on the fifth day of admission.

On the third day after degarelix administration, the patient exhibited clinical deterioration with marked prostration, accompanied by laboratory evidence of hyperuricemia (13 mg/dL), hyperphosphatemia (6.5 mg/dL), hyperkalemia (5.5 mmol/L), and acute kidney injury, classified as Acute Kidney Injury Network (AKIN) stage III (creatinine = 5.0 mg/dL) (Table 1).

**Table 1:** Evolution of laboratory parameters during admission and before and after degarelix administration. INR: international normalized ratio; APTT: activated partial thromboplastin time; AST: aspartate aminotransferase; ALT: alanine aminotransferase; GGT: gamma-glutamyl transferase; BUN: blood urea nitrogen; PSA: prostate-specific antigen.

	Reference values	Pre-hormonal therapy	3 days after hormonal therapy	5 days after hormonal therapy
Hematology				
Hemoglobin (g/dL)	13.0-17.0	12.5	11.1	11.0
Hematocrit (%)	40-50	36.3	32.8	33.3
Mean cell volume (fL)	78-96	85.8	88.9	90.7
Mean corpuscular hemoglobin (pg)	27-32	29.6	30.1	30.0
Leukocytes (x10^9/L)	4.0-10.0	11.8	13.4	12.9
Neutrophils (x10^9/L)	1.8-6.9	8.9	10.7	10.6
Lymphocytes (x10^9/L)	1.2-3.3	1.8	1.5	1.4
Platelet count (x10^9/L)	150-410	384	357	324
Erythrocyte sedimentation rate (mm)	<13	103		
Coagulation				
Prothrombin time (sec)	9.7-11.8	13.5	14.7	14.8
INR	<1.2	1.3	1.4	1.5
APTT (sec)	20.6-29.5	24.7	27.9	26.4
Biochemistry				
AST (U/L)	<40	111	225	1126
ALT (U/L)	<40	47	60	213
Alkaline phosphatase (U/L)	40-130	435	612	652
GGT (UI/L)	<60	462	680	595
Total bilirubin (mg/dL)	<1.2	1.02	1.3	1.3
Potassium (mmol/L)	3.5-5.1	4.1	5.5	4.6
Phosphorus (mg/dL)	2.5-4.5	4	6.5	5
Total calcium (mg/dL)	8.8-10.2	8.5	8.5	8.8
Uric acid (mg/dL)	3.4-7	8,2	13	<0.2
Lactate dehydrogenase (U/L)	132-225	3469	4730	14613
Creatinine (mg/dL)	0.7-1.2	1.14	5.1	2.9
BUN (mg/dL)	<50	47	130	141
Total PSA (ng/mL)	<4.5	74.2		

Based on these findings, a diagnosis of TLS secondary to hormonal therapy was established. Aggressive intravenous hydration was initiated, along with rasburicase (0.2 mg/kg) and correction of electrolyte abnormalities.

It was determined that renal replacement therapy was not required at that stage, given the improvement of hyperuricemia after rasburicase administration and preserved diuresis following intensified hydration.

Despite supportive measures, the patient experienced progressive clinical deterioration with severe hepatic failure and marked encephalopathy. Given the absence of feasible oncologic treatment options, the high burden of plurimetastatic disease, and the overall poor prognosis, a multidisciplinary assessment with the oncology and intensive care teams determined that further invasive interventions would not be beneficial. The patient, unfortunately, died 17 days after the onset of TLS.

## Discussion

TLS occurs most frequently in hematologic malignancies due to their high proliferative rate and sensitivity to cytotoxic therapy [8]. TLS is uncommon in solid tumors, but it has been documented in metastatic prostate cancer.

Several risk factors have been associated with the development of TLS, including elevated LDH and uric acid levels, chronic kidney disease, extensive metastatic burden, and tumors with high proliferative activity. Recognizing these factors is crucial, as their presence may warrant prophylactic measures prior to initiating antitumor therapy, including vigorous intravenous hydration to promote renal excretion, the use of xanthine oxidase inhibitors (e.g., allopurinol) or urate oxidase agents (e.g., rasburicase) to prevent hyperuricemia, and frequent monitoring of electrolytes and renal function [1].

In addition to primary prevention, early diagnosis and prompt treatment of TLS are critical to reducing morbidity and preventing life-threatening complications, such as severe electrolyte disturbances, acute kidney injury, and cardiac arrhythmias.

In this case, several factors likely contributed to the development of TLS. The patient presented with extensive metastatic disease and markedly elevated LDH, both recognized predictors of TLS. Furthermore, the histopathological findings suggested tumor dedifferentiation, indicating increased cellular turnover [4]. Degarelix induces rapid suppression of androgen signaling, potentially triggering abrupt tumor cell apoptosis in highly proliferative metastatic disease, precipitating TLS [9-10].

To date, 10 cases of TLS associated with prostate cancer have been reported in the literature, including three spontaneous and seven therapy-related, all occurring in the context of metastatic disease [4]. Among the therapy-related cases, only one has previously been attributed to hormonal therapy with goserelin acetate, in which the patient required dialysis and intensive intravenous hydration, but despite these measures, the clinical course was fatal [4,7].

The literature consistently shows that TLS occurring after initiation of hormonal therapy is associated with extremely poor outcomes. In all documented treatment-related TLS cases in prostate cancer, mortality was universal despite timely recognition and aggressive supportive management [4].

The present report represents the second documented case of TLS secondary to hormonal therapy and the first associated specifically with the GnRH antagonist degarelix.

## Conclusions

TLS secondary to hormonal therapy, although rare, is a potential complication in metastatic prostate cancer, particularly in the presence of high tumor burden and rapid treatment response. Clinicians should maintain a high index of suspicion for TLS when initiating systemic therapy, including hormonal agents, in patients with extensive metastatic disease and elevated LDH levels. This case reinforces the importance of clinical vigilance, careful risk stratification before starting hormonal therapy, and close biochemical monitoring in high-risk patients.
